# Serum vascular endothelial growth factor load and interleukin-6 in cancer patients

**DOI:** 10.1054/bjoc.1999.1115

**Published:** 2000-06

**Authors:** K J O'Byrne, N Dobbs, A L Harris

**Affiliations:** University Department of Oncology, Leicester Royal Infirmary, LE1 5WW, UK; Imperial Cancer Research Fund Medical Oncology Unit, Churchill Hospital, Oxford, OX3 7LJ, UK


*British Journal of Cancer* (2000) 82(**11**), 1895–1896
© 2000 Cancer Research Campaign
DOI: 10.1054/ bjoc.1999.1115, available online at http://www.idealibrary.com on IDEAL®

Letter to the Editor

### Serum vascular endothelial growth factor load and interleukin-6 in cancer patients

**Sir**

In a recent study published in the *British Journal of Cancer*, Salgado and colleagues demonstrated a correlation between serum IL-6 levels, serum VEGF levels and platelet count in patients with advanced cancer. Patients with a thrombocytosis had median VEGF serum concentrations × 3.2, and median IL-6 serum levels × 5.8, higher than other patients. Growth factors and cytokines such as IL-6 may affect both platelet number and function, including the capacity of platelets to store angiogenic factors such as VEGF. This is supported by the observation that IL-6 levels were also found to correlate with the VEGF load per platelet ($VEGF^{PLT}$) (Salgado et al, 1999).

We have recently demonstrated a close correlation between platelet counts and serum VEGF levels in patients with renal cell cancer (RCC). Platelet counts and VEGF levels ≥ the median for the group indicated a poor outcome (O'Byrne et al, 1999). In reply to our report Gunsillius and colleagues recommended we calculate the $VEGF^{PLT}$ in the patients in our study and assess its impact on outcome (Gunsilius et al, 1999). The mean $VEGF^{PLT}$ value for the whole group was 1.29 pg/$10^6$ platelets (range 0.13–4.06). This is similar to that found by Salgado and colleagues where the mean value was 1.37 pg/$10^6$ (range 0.16–3.64). Patients with $VEGF^{PLT}$ levels ≥ median had a significantly worse outcome than those with lower levels ($P$ = 0.025, Figure 1). Whilst there was a trend for $VEGF^{PLT}$ concentrations to be higher in patients with platelet counts ≥ median value for the group this was not significant ($P$ = 0.174).

The prognostic significance of $VEGF^{PLT}$ may be due to high local release of VEGF by platelets activated in solid tumours potentially leading to enhanced angiogenesis. The activation of platelets may occur through their adherence to tumour-related endothelium or by their extravasation through hyperpermeable microvessels with subsequent exposure to subendothelial matrix proteins (Pinedo et al, 1998; O'Byrne et al, 1999). The relatively wide range of $VEGF^{PLT}$ levels seen in these studies is likely to reflect, at least in part, the activity of cytokines such as IL-6 overexpressed in RCC. The correlation with $VEGF^{PLT}$ provides an explanation for the observation that elevated serum IL-6 levels are associated with a poor outcome in solid tumours including RCC (Dosquet et al, 1997). These results also lend support to the contention of Pinedo and colleagues of an important role for platelets in the process of tumour-related angiogenesis (Pinedo et al, 1998).

*KJ O'Byrne[1], N Dobbs[2], AL Harris[2]*
*[1]University Department of Oncology, Leicester Royal Infirmary, LE1 5WW, UK; [2]Imperial Cancer Research Fund Medical Oncology Unit, Churchill Hospital, Oxford, OX3 7LJ, UK*

**REFERENCES**

Dosquet C, Courdert MC, Lepage E, Cabane J and Richard F (1997) Are angiogenic factors, cytokines, and soluble adhesion molecules prognostic factors in patients with renal cell carcinoma? *Clin Cancer Res* **3**: 2451–2458

Gunsilius E, Petzer AL and Gastl G (1999) Vascular endothelial growth factor platelet counts and renal cancer. *Lancet* **353**: 2247

Pinedo HM, Verheul HMW, D'Amato RJ and Folkman J (1998) Involvement of platelets in tumour angiogenesis? *Lancet* **352**: 1775–1777

O'Byrne KJ, Dobbs N, Propper D, Smith K and Harris AL (1999) Vascular endothelial growth factor, platelet counts, and prognosis in renal cancer. *Lancet* **353**: 1494–1495

Salgado R, Vermeulen PB, Benoy I, Weytjens R, Huget P, Van Marck E and Dirix LY (1999) Platelet number and interleukin-6 correlate with VEGF but not with bFGF serum levels of advanced cancer patients. *Br J Cancer* **80**: 892–897

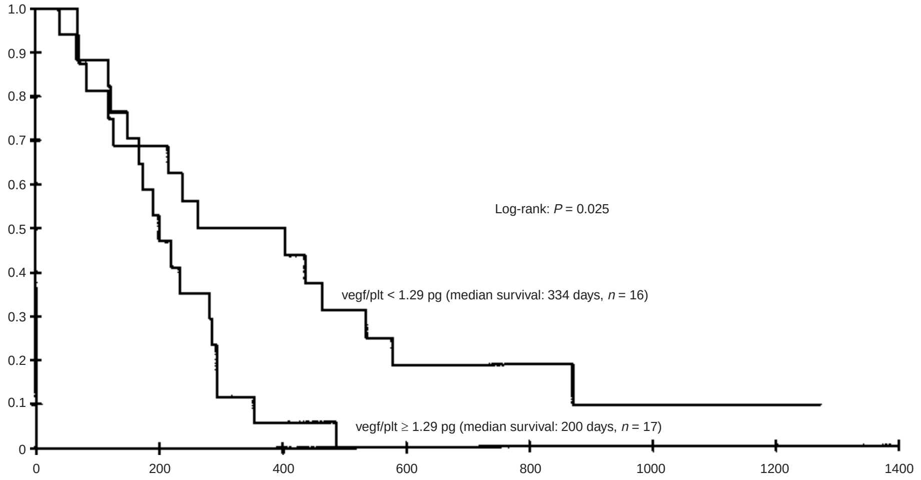

**Figure 1** Survival difference between patients with vegf/platelet ratio above and below the group median (1.29 pg) prior to treatment

